# Action of Various Diuretics

**Published:** 1880-07

**Authors:** 


### ACTION OF VARIOUS DIURETICS.

Dr. Maurel gives the result of his experiments (*Bull. Gén. de Thérap.*, Nos. 5 and 6, 1880) as follows: 1. Nitrate of potassium, uncertain as to the quantity of liquid, augments the solid materials of the urine to a notable degree. The most active doses are a drachm to a drachm and a half. 2. Chlorate of potassium, less active with respect to the augmentation of solids, increases the fluids of the urine to a greater degree. 3. Acetate of potassium is uncertain, as to the quantity of both solids and and fluids. 4. Iodide of potassium, far from being a diuretic, even seems to diminish the quantity of urine. 5. Salicylate of sodium, uncertain as to the quantity of liquid, isncreases the solid constituents of the urine. 6. Of three vegetable substances experimented upon,—squill, colchicum, and digitalis,—the latter alone is a real diuretic. It augments at the same time the quantity of both solids and fluids. Dr. Maurel gives it as his opinion that no diuretic acts when the system is in a febrile condition: this must be modified before diuresis can occur.

